# Continuous Glucose Monitoring in Insulin-Treated Older Adults With Diabetes and Alzheimer Disease and Related Dementias

**DOI:** 10.1001/jamanetworkopen.2025.41939

**Published:** 2025-12-02

**Authors:** Pareeta Kotecha, Steven M. Smith, William T. Donahoo, Steven T. DeKosky, Jiang Bian, Jingchuan Guo

**Affiliations:** 1 Department of Pharmaceutical Outcomes and Policy, College of Pharmacy, University of Florida, Gainesville; 2 Center for Drug Evaluation and Safety, University of Florida, Gainesville; 3 Division of Endocrinology, Diabetes and Metabolism, College of Medicine, University of Florida, Gainesville; 4 McKnight Brain Institute, University of Florida, Gainesville; 5 Department of Neurology, College of Medicine, University of Florida, Gainesville; 6 Department of Biostatistics & Health Data Science, School of Medicine, Indiana University, Indianapolis; 7 Regenstrief Institute, Indianapolis, Indiana

## Abstract

**Importance:**

Cognitive impairment and glycemic control have a bidirectional association. Understanding the impact of continuous glucose monitoring (CGM) vs self-monitoring of blood glucose (SMBG) is important for treating older adults with Alzheimer disease and related dementias (ADRD) and diabetes that is treated with insulin.

**Objective:**

To compare the risks of glycemic outcomes and related adverse events between CGM users and prevalent SMBG users in insulin-treated older adults with ADRD and diabetes.

**Design, Setting, and Participants:**

This retrospective, prevalent–new user cohort study utilized a nationwide, 15% random sample of Medicare claims data (Parts A, B, and D) from January 2016 to December 2020 to ensure balanced cohorts. Insulin-treated older adults (≥66 years) with ADRD and diabetes were included. Individuals in hospice care or with a professional CGM use were excluded. Analysis was carried out from August 2023 to December 2024.

**Exposure:**

Therapeutic CGM use vs prevalent SMBG use.

**Main Outcomes and Measures:**

Primary outcomes included hypoglycemia hospitalizations, hyperglycemia crisis, and all-cause mortality; falls and all-cause hospitalizations were secondary outcomes. Upper respiratory tract infections served as a negative control outcome. A 1:1 propensity score matching with a 0.25 caliper was carried out for confounding control. Cox proportional hazards models were used for outcome analysis.

**Results:**

In this cohort of 2022 insulin-treated older adults with diabetes and ADRD (1011 CGM users and 1011 SMBG users; mean [SD] age, 76.4 [6.7] years; 1133 female [56.0%]), CGM use was significantly associated with lower risk of all-cause hospitalization (hazard ratio [HR], 0.86; 95% CI, 0.76-0.96) and mortality (HR, 0.57; 95% CI, 0.48-0.67) compared with SMBG use. CGM use had lower point estimates for hypoglycemia hospitalization (HR, 0.66; 95% CI, 0.40-1.08) and falls (HR, 0.86; 95% CI, 0.68-1.08) and higher point estimates for hyperglycemia crisis (HR, 1.38; 95% CI, 0.99-1.94) vs SMBG use, but these findings were not significant. Exploratory subgroup analyses showed varied benefits. The negative control outcome showed no significant differences across analyses.

**Conclusions and Relevance:**

In this cohort study of insulin-treated older adults with ADRD and diabetes, CGM use was associated with improved long-term clinical outcomes. Pragmatic (ie, evaluating the effectiveness of healthcare interventions in everyday settings) trials are needed to validate these findings and to assess the feasibility of CGM use in this population.

## Key Points

**Question:**

Is the use of continuous glucose monitoring (CGM) associated with improved glycemic outcomes and related adverse events in insulin-treated older adults with Alzheimer disease and related dementias (ADRD) and diabetes?

**Findings:**

This cohort study of 1011 CGM users and 1011 prevalent SMBG users who were propensity score–matched found that CGM use was associated with a significantly lower risk of all-cause hospitalization and all-cause mortality, while associations with glycemic outcomes varied.

**Meaning:**

This study highlights the potential long-term benefits of CGM in insulin-treated older adults with diabetes and ADRD and the need for clinical trials in this population.

## Teaser

This cohort study compares the risk of glycemic outcomes and adverse events among insulin-treated adults with diabetes and Alzheimer disease and related dementias who use either continuous glucose monitoring (CGM) or self-monitoring of blood glucose.

## Introduction

The US is experiencing a substantial demographic shift, with the older adult population growing rapidly.^1^ Older adults (≥65 years) face an increased risk of developing chronic health conditions such as diabetes and Alzheimer disease and related dementias (ADRD; ie, impairment of memory, thought processes, and functioning).^2^ Additionally, the prevalence of mild cognitive impairment or dementia among older adults with diabetes ranges from 18% to 39% in the US.^2,3,4^

The presence of coexisting diabetes and ADRD increases the risk of adverse health outcomes.^5^ Furthermore, the association of glycemic control with cognitive impairment is bidirectional; episodes of hypoglycemia or hyperglycemia can lead to worsening cognition, and cognitive impairment could increase the risk of hypoglycemia and hyperglycemia, particularly in patients with insulin-treated diabetes, exacerbating this bidirectional association.^5^ Factors such as poor nutrition, insulin dosing, and difficulty recognizing and responding to symptoms of hypoglycemia and hyperglycemia increase the risk of poor glycemic control in this population.^5,6,7^

Current American Diabetes Association (ADA) guidelines recommend a preventative approach to avoid hypoglycemia for older adults with cognitive impairment by targeting a hemoglobin A_1C_ level of less than 8.0% in those with mild to moderate impairment and prioritizing hypoglycemia avoidance over strict glycemic targets in those with moderate to severe impairment.^8^ The guidelines for diabetes treatment and older adults also emphasize considering cognitive impairment when prescribing glucose-lowering medications (ie, deintensification or realignment of therapy by diabetic endocrinologists).^9,10,11^ However, simply raising hemoglobin A_1C_ targets in this population may not ensure safe glycemic control because inadequate monitoring could increase the risk of hyperglycemia. Conversely, there is evidence of overtreatment despite higher targets, which may heighten the risk of severe hypoglycemia, particularly among those with hypoglycemia unawareness.^12,13,14,15^ ADA guidelines endorse continuous glucose monitoring (CGM) in older adults with type 1 and type 2 diabetes (T1D and T2D) and suggest exploring its use in those with cognitive impairment who struggle with self-monitoring of blood glucose (SMBG).^8,9^

Additionally, patients with ADRD are at an increased risk of falls. This risk is amplified when hypoglycemia or hyperglycemia induces autonomic dysfunction.^16,17,18,19^ It is crucial to identify and address modifiable risk factors to prevent falls in the aging population since the risk of fall-related mortality is higher in older adults.^20^ Additionally, poor glycemic control is associated with fatal outcomes, particularly in those who are frail or have multiple health conditions such as ADRD and diabetes.^21,22,23,24,25^

Our pilot retrospective study^26^ in the University of Florida Health System found that CGM use in insulin-treated patients with diabetes and mild cognitive impairment or ADRD was associated with lower odds of all-cause mortality, while hypoglycemia-related hospitalizations were associated with increased the odds of mortality risk. However, evidence on CGM use that is generalizable to insulin-treated older adults with diabetes and ADRD remains limited. Therefore, we aimed to compare the outcomes of therapeutic CGM users (therapeutic and/or nonadjunctive CGM are approved for clinical decision-making without confirmatory SMBG; ie, even if a patient uses both, decisions will be made based on CGM readings) vs prevalent SMBG users in insulin-treated Medicare beneficiaries with coexisting diabetes and ADRD.

## Methods

### Data Source, Study Design, and Study Population

This cohort study utilized administrative claims data from a 15% random sample of nationwide Medicare fee-for-service beneficiaries with Part A, Part B, and Part D from January 1, 2016, to December 31, 2020. This study was exempt from review and the requirement of informed consent by the University of Florida institutional review board under Category 4 (secondary research without consent) of 45 CFR §160 and §164. This study followed the Strengthening the Reporting of Observational Studies in Epidemiology (STROBE) reporting guideline.

This was a retrospective, prevalent-new user cohort study, with prevalent users being SMBG users at baseline and new users being therapeutic CGM initiators during the study period. We included insulin-treated patients with diabetes and ADRD. We used the Centers for Medicare and Medicaid Services Chronic Condition Warehouse variables to identify patients with both ADRD and diabetes, and patients were required to have a claim of insulin at baseline. Nonusers without evidence of SMBG at baseline and beneficiaries with a *Current Procedural Terminology* claim for professional CGM or ProCGM were excluded. ProCGM users were excluded because it was presumed to include only short-term users of CGM and because the Centers for Medicare and Medicaid Services reimbursement criteria require a Healthcare Common Procedure System code. Additionally, we excluded patients in hospice care or patients with a CGM claim in 2020 to allow for at least 1 year of follow-up after initiation.

We applied the inclusion and exclusion criteria twice to ensure robust and comparable cohort selection. First, we assessed eligibility between January 1 and December 31, 2016, to identify patients meeting the eligibility criteria to receive CGM or SMBG. From this cohort, patients were screened for CGM claims between January 1, 2017, and December 31, 2019. The date of CGM initiation was defined as the index date (ie, follow-up start date). For those who did not initiate CGM, we assigned an index date if they had an SMBG claim in the initial eligibility period (see the Exposure subsection for further details). Second, the patients had to meet the eligibility criteria and complete medical and pharmacy information (−365 days) after both groups had an index date.

This 2-step approach ensured that both CGM and SMBG users had complete data and a comparable assignment of index dates. Applying the criteria only once could cause disproportionate distribution of index dates between the groups, misaligning the match.

### Outcomes of Interest

The primary study outcomes included hypoglycemia hospitalizations, hyperglycemic crises, and all-cause mortality. Falls and all-cause hospitalizations were secondary outcomes. A negative control outcome was used to assess potential unmeasured confounding or selection bias. These outcomes are subject to similar sources of bias as the primary outcomes but are not plausibly affected by CGM use; therefore, upper respiratory tract infections were considered as a negative control outcome.^27,28,29^

Hypoglycemia hospitalizations, hyperglycemic crises, falls, and upper respiratory tract infections were identified using *International Classification of Diseases, Ninth Revision (ICD-9)* and *International Statistical Classification of Diseases and Related Health Problems, Tenth Revision (ICD-10)* codes.^30,31,32,33^ Hypoglycemia hospitalizations were defined as an inpatient claim with the code for hypoglycemia with or without coma. Hyperglycemic crisis was defined as an emergency or inpatient claim of diabetic ketoacidosis or hyperosmolar hyperglycemic syndrome. The first occurrence of the outcomes after the index date at the primary or secondary diagnosis level was considered as the date of the respective event. Time to all-cause hospitalization was captured as the first record in the inpatient file after the index date, and all-cause mortality was captured from the Master Beneficiary Summary File.^34^

### Exposure of Interest

This was a prevalent-new user study. Patients were identified as therapeutic CGM users if they had a durable medical equipment vendor claim for therapeutic CGM supplies (Healthcare Common Procedure System code K0553 or K0554) anytime from January 1, 2017, through December 31, 2019, to allow for at least 1 year of follow-up.^35,36^ The first claim of CGM was marked as the index date for the user. Patients who did not initiate CGM were classified as prevalent SMBG users if they had at least 1 claim for blood glucose test strips between January 1 and December 31, 2016, and an additional claim in the baseline period after an index date was assigned. SMBG users were identified using the Healthcare Common Procedure System code A4253 for blood glucose test strips.

To minimize the possibility of immortal time bias, we used prescription time matching to assign an index date to prevalent SMBG users; this ensured a clearly defined follow-up start date, allowing for appropriate comparison with CGM users and reducing the risk of misclassifying periods during which the outcome could not occur.^37,38^ Prescription time matching ensured that CGM users and SMBG users had similar recorded days since ADRD or cognitive impairment. This was done using CGM index dates as reference, and days to initiation from the first diagnosis of ADRD or cognitive impairment were calculated for CGM users. SMBG users were assigned an index date based on a comparable time from the first recorded diagnosis of ADRD or cognitive impairment, ensuring a similar trend for the distribution of the index between the groups. Because it is difficult to determine the severity of cognitive impairment from claims data, aligning the index dates based on time from diagnosis could help account for the cognitive impairment severity between the groups due to the progressive nature of the condition.^39^ This approach is particularly important given that ADA guidelines recommend glycemic control based on cognitive impairment severity.^8^ The process is depicted in eFigure 1 and eFigure 2 in Supplement 1.

### Covariates

Our analysis considered a wide range of covariates to control for potential confounding. We collected the beneficiaries’ sociodemographic and clinical information during the lookback period of 365 days from the index date. Demographic covariates included age, sex, race and ethnicity (Asian and Pacific Islander, American Indian or Alaska Native, non-Hispanic Black, non-Hispanic White, other [any race or ethnicity not otherwise specified], and unknown), region, and metropolitan area. We also accounted for several comorbidities and comedications, and use of insulin pumps, as well as diabetes and ADRD-related events. Lastly, we included health care utilization, markers for healthy behavior (vaccination status),^40^ frailty index, and the Charlson Comorbidity Index (CCI).^41,42,43^

### Statistical Analysis

Data analysis occurred from August 2023 to December 2024. All analyses were performed using SAS version 9.4. (SAS Institute).

#### Primary Analysis

To mitigate confounding, we estimated propensity scores for CGM initiation vs SMBG use using a multivariable logistic regression model. Treatment status (CGM vs SMBG) was the dependent variable, and baseline covariates reflecting demographic characteristics, comorbid conditions, medication use, and health care utilization were included as independent variables. Each individual’s propensity score represented the estimated probability of initiating CGM, conditional on observed characteristics. A 1:1 greedy propensity score matching without replacement was utilized, wherein each exposed participant was matched with the closest unexposed participant within the specified standard caliper of 0.25.^44,45^ A successful match was considered when an absolute standardized mean difference (aSMD) of covariates less than 0.1 was achieved.^44^ Continuous data are presented as mean (SD), and categorical data are presented as number (percentage).

We employed Cox proportional hazards models in the matched cohort to analyze our primary clinical outcomes and negative control events. Patients were followed until the first event of interest after the index date, death, end of continuous enrollment, or December 31, 2020, whichever came first. Variables that had an aSMD greater than 0.1 after propensity score matching were added to the Cox model. Prior to model fitting, we confirmed that the convergence status and proportionality assumption were satisfied. Statistical significance was determined as a 95% CI that did not include 1.0.

#### Sensitivity and Subgroup Analyses

We carried out sensitivity analyses to address the competing risk of all-cause mortality; a Cox proportional hazards model with the Fine and Gray method was used to estimate the adjusted subdistribution hazard ratio (HR) for hypoglycemia hospitalizations, hyperglycemia crisis, falls, and all-cause hospitalizations.^46^ Additionally, we assessed the 3-month hypoglycemia hospitalizations and hyperglycemia crisis in sensitivity analyses. Subgroup analyses assessed the exposure-outcome association by subgroups, including age, sex, race and ethnicity, T2D, and frailty score (prefrail: 0.15-0.24; mildly frail: 0.25-0.34; moderate to severely frail: ≥0.35),^41,47^ and CCI score (moderate: 3-4; severe: ≥5).^43,48^ Subgroup analyses were exploratory and hypothesis-generating.

## Results

**Figure 1.**
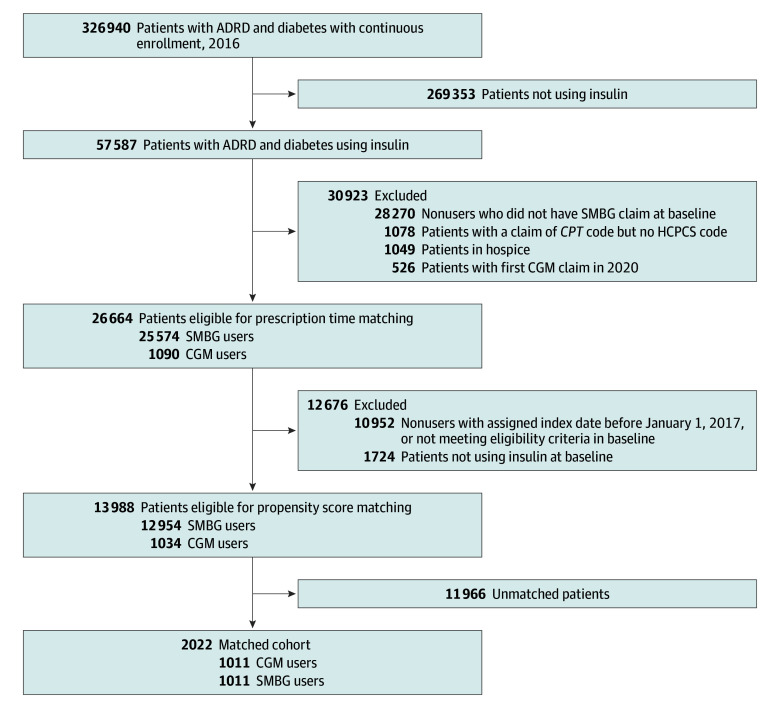
Study Cohort Selection ADRD indicates Alzheimer disease and related dementias; CGM, continuous glucose monitoring; *CPT*, *Current Procedural Terminology*; HCPCS, Healthcare Common Procedure Coding System; SMBG, self-monitoring of blood glucose.

A total of 13 988 insulin-treated older adults with diabetes and ADRD were included before propensity score matching (Figure 1), with 1034 CGM users and 12 954 SMBG users. CGM users were generally younger (mean [SD] age, 76.3 [6.5] years vs 78.8 [7.2] years), had a higher percentage of males (465 participants [45.0%] vs 4584 participants [35.4%]), non-Hispanic White participants (820 participants [79.3%] vs 7843 participants [60.5%]), participants with T1D (460 participants [44.5%] vs 2479 participants [19.1%]), and participants who lived in metropolitan areas (906 participants [87.6%] vs 10 774 participants [83.2%]) compared with SMBG users before matching. CGM users also had higher proportions of patients using rapid-acting insulins (873 participants [84.4%] vs 5969 participants [46.1%]), patients with hypoglycemia hospitalizations (29 participants [2.8%] vs 213 participants [1.6%]) and hyperglycemia crisis (56 participants [5.4%] vs 369 participants [2.8%]), a higher mean (SD) number of endocrinologist visits (0.2 [0.9] visits vs 0.04 [0.4] visits), and a higher proportion of patients with vaccinations (594 patients [57.5%] vs 5369 patients [41.4%]) at baseline compared with SMBG users. After matching, 2022 patients were included (mean [SD] age, 76.4 [6.7] years; 1133 female [56.0%]; 209 Hispanic [10.3%]; 144 non-Hispanic Black [7.1%]; 1578 non-Hispanic White [78.0%]), with 1011 CGM users and 1011 SMBG users. Matching resulted in a balance between the CGM and SMBG groups with an aSMD less than 0.1 for all variables (Table 1 and eFigure 3 in Supplement 1). The overall median (IQR) follow-up in the matched cohort was 701 (419-780) days, 639 (441-818) days for the CGM group, and 721 (350-772) days for the SMBG group.

**Table 1.** Baseline Characteristics Before and After Propensity Score Matching

Variable	Participants, No. (%)					
	Before matching			After matching (1:1)		
	CGM user (n = 1034)	SMBG user (n = 12 954)	SMD	CGM user (n = 1011)	SMBG user (n = 1011)	SMD
Demographic characteristics						
Age, mean (SD), y	76.3 (6.5)	78.8 (7.2)	−0.36^a^	76.3 (6.5)	76.4 (6.8)	−0.01
Sex						
Female	569 (55.0)	8370 (64.6)	0.20^a^	559 (55.3)	574 (56.8)	0.03
Male	465 (45.0)	4584 (35.4)		452 (44.7)	437 (43.2)	
Race and ethnicity						
Asian and Pacific Islander	24 (2.3)	371 (2.9)	0.03	24 (2.4)	26 (2.6)	0.01
American Indian or Alaska Native	≤11	40 (0.3)	0.02	≤11	≤11	−0.02
Hispanic	99 (9.1)	2830 (21.8)	0.34^a^	99 (9.8)	110 (10.9)	0.04
Non-Hispanic Black	72 (6.9)	1721 (13.3)	0.21^a^	70 (6.9)	74 (7.3)	0.02
Non-Hispanic White	820 (79.3)	7843 (60.5)	−0.42^a^	800 (79.1)	778 (76.9)	−0.05
Other^b^	≤11	114 (0.9)	−0.01	≤11	Suppressed^c^	0.05
Unknown	≤11	35 (0.3)	−0.05	≤11	≤11	0.00
Region						
Northeast	145 (14.02)	1876 (14.5)	0.01	141 (13.9)	158 (15.6)	0.05
Midwest	155 (14.9)	2059 (15.9)	0.02	150 (14.8)	150 (14.8)	0.00
West	Suppressed^c^	1404 (10.8)	−0.03	118 (11.7)	116 (11.5)	−0.01
South	610 (58.9)	7585 (58.5)	−0.01	602 (59.5)	587 (58.1)	−0.03
Unknown	≤11	30 (0.2)	0.01	NA	NA	NA
Metropolitan city	906 (87.6)	10 774 (83.2)	−0.13^a^	887 (87.7)	884 (87.4)	−0.01
Type 1 diabetes	460 (44.5)	2479 (19.1)	−0.57^a^	443 (43.8)	438 (43.3)	−0.01
Comorbidities						
Obesity	451 (43.6)	5407 (41.7)	−0.04	442 (43.7)	457 (45.2)	0.03
Hypothyroidism	599 (57.9)	6707 (51.8)	−0.12^a^	586 (57.9)	608 (60.1)	0.04
Hypertension^c^	>1008 (>97.5)	>12 208 (>94.2)	0.02	>986 (>97.5)	>982 (>97.1)	0.02
Hyperlipidemia^c^	>1008 (>97.5)	>12 208 (>94.2)	0.02	>986 (>97.5)	>982 (>97.1)	0.03
Atrial fibrillation	296 (28.6)	3979 (30.7)	0.05	293 (28.9)	290 (28.7)	−0.01
Congestive heart failure	659 (63.7)	9361 (72.3)	0.19^a^	648 (64.1)	635 (62.8)	−0.03
Stroke	467 (45.2)	6347 (49.0)	0.08	458 (45.3)	473 (46.8)	0.01
Acute myocardial infarction	165 (16.3)	2110 (16.3)	0.01	161 (15.9)	165 (16.3)	0
Benign prostatic hyperplasia	341 (32.9)	3432 (26.5)	−0.14	330 (32.6)	336 (33.2)	0.01
Depression	763 (72.8)	9891 (76.3)	0.06	747 (73.9)	760 (75.2)	0.03
Anxiety	291 (28.1)	3522 (27.2)	−0.02	284 (28.1)	286 (28.3)	0.04
Schizophrenia	27 (2.6)	920 (7.1)	0.21^a^	27 (2.7)	26 (2.6)	0.02
Breast cancer	78 (7.5)	969 (7.5)	0.00	77 (7.6)	86 (8.5)	0.03
Lung cancer	28 (2.7)	255 (1.9)	−0.05	27 (2.7)	30 (2.9)	−0.01
Colon cancer	40 (3.9)	608 (4.7)	0.04	40 (3.9)	40 (3.9)	−0.03
Prostate cancer	75 (7.2)	856 (6.6)	−0.03	73 (7.2)	69 (6.8)	0.03
Endometrial cancer	14 (1.3)	207 (1.6)	0.02	14 (1.4)	14 (1.4)	0.01
History of chronic kidney disease	1008 (97.5)	12 208 (94.2)	−0.16^a^	986 (97.5)	982 (97.1)	0.03
History of brain hemorrhage	918 (88.8)	11 418 (88.1)	−0.02	900 (89.0)	893 (88.3)	0.00
Parkinson disease	46 (4.4)	755 (5.8)	0.06	45 (4.4)	42 (4.1)	−0.01
Epilepsy	58 (5.6)	708 (5.5)	−0.01	57 (5.6)	58 (5.7)	0.03
Posttraumatic stress disorder	18 (1.7)	90 (0.7)	−0.10	16 (1.6)	19 (1.9)	0.02
Obsessive compulsive disorder	≤11	41 (0.3)	−0.03	≤11	≤11	0.00
Alcohol use disorder	29 (2.8)	227 (1.8)	−0.07	28 (2.8)	25 (2.5)	−0.02
Traumatic brain injury	18 (1.7)	238 (1.8)	0.01	17 (1.7)	17 (1.7)	0.00
Anemia	935 (90.4)	11 902 (91.9)	0.05	918 (90.8)	924 (91.4)	0.02
Osteoporosis	404 (39.1)	5006 (38.6)	−0.01	396 (39.2)	398 (39.4)	−0.02
Sleep disorder	407 (39.4)	3766 (29.1)	−0.22^a^	393 (38.9)	390 (38.6)	−0.01
Rheumatoid arthritis or osteoarthritis	891 (86.7)	11 264 (86.9)	0.02	874 (86.4)	872 (86.2)	−0.02
Substance use disorder	118 (11.4)	1376 (10.6)	−0.02	113 (11.2)	107 (10.6)	−0.02
Hearing impairment	156 (15.1)	1353 (10.4)	−0.14^a^	152 (15.0)	147 (14.5)	−0.01
Visual impairment	25 (2.4)	399 (3.1)	0.04	24 (2.4)	29 (2.9)	0.03
Glaucoma	418 (40.4)	5166 (39.88)	−0.01	409 (40.4)	428 (42.3)	0.04
Asthma	316 (30.6)	3964 (30.6)	0.00	309 (30.6)	302 (29.9)	−0.02
Chronic obstructive pulmonary disorder	546 (52.8)	7538 (58.2)	0.11^a^	535 (52.9)	531 (52.5)	−0.01
Comedications						
Long-acting insulins	859 (83.1)	10 562 (81.5)	−0.04	850 (84.1)	878 (86.8)	0.08
Rapid-acting insulins	873 (84.4)	5969 (46.1)	−0.88^a^	851 (84.2)	859 (85.0)	0.02
Mixed insulins	88 (8.5)	2187 (16.9)	0.26^a^	87 (8.6)	78 (7.7)	−0.03
Intermediate-acting insulins	32 (3.1)	454 (3.5)	0.03	32 (3.2)	32 (3.2)	0.00
Short-acting insulins	40 (3.9)	795 (6.1)	0.10^a^	40 (3.9)	42 (4.1)	0.01
Thiazolidinediones	24 (2.3)	370 (2.9)	0.03	23 (2.3)	16 (1.6)	−0.05
Antidepressants	584 (56.5)	7536 (58.2)	0.03	573 (56.7)	560 (55.4)	−0.03
Oral steroids	477 (46.1)	5285 (40.8)	−0.11^a^	465 (45.9)	468 (46.3)	0.01
Antidementia	258 (24.9)	4043 (31.2)	0.14^a^	252 (24.9)	252 (24.9)	0.00
Antipsychotics	51 (4.9)	1077 (8.3)	0.14^a^	51 (5.0)	57 (5.6)	0.03
Statins	867 (83.8)	10 031 (77.4)	−0.16^a^	846 (83.7)	848 (83.9)	0.01
Nonsteroidal anti-inflammatory drugs	217 (21.0)	2847 (21.9)	0.02	213 (21.1)	214 (21.2)	0.00
Dipeptidyl peptidase-4 inhibitors	93 (8.9)	2176 (16.8)	0.23^a^	93 (9.2)	100 (9.9)	0.02
Calcium channel blockers	434 (42.0)	5629 (43.4)	0.03	422 (41.7)	437 (43.2)	0.03
Opioids	367 (35.5)	3767 (29.1)	−0.14^a^	357 (35.3)	349 (34.5)	−0.02
Diuretics	544 (52.6)	6878 (53.1)	0.01	531 (52.5)	543 (53.7)	0.02
β-blockers	653 (63.1)	8151 (62.9)	0.00	640 (63.3)	664 (65.7)	0.05
Angiotensin converting enzyme inhibitors	344 (33.3)	4373 (33.8)	0.01	339 (33.5)	370 (36.6)	0.06
Angiotensin receptor blockers	409 (39.6)	4418 (34.1)	−0.11^a^	396 (39.2)	394 (38.9)	0.00
Anticoagulants	214 (20.7)	2721 (21.0)	0.01	212 (21.0)	209 (20.7)	−0.01
Sulfonylureas	91 (8.8)	2361 (18.2)	0.28	91 (9.0)	94 (9.3)	0.01
Antiplatelets	49 (4.5)	519 (3.9)	−0.04	45 (4.5)	46 (4.5)	0.00
Glucagon-like peptide-1 receptor agonist	106 (10.3)	724 (5.6)	−0.17^a^	104 (10.3)	107 (10.6)	0.01
Sodium-glucose cotransporter inhibitor	34 (3.3)	352 (2.7)	−0.03	33 (3.3)	24 (2.4)	−0.05
Meglitinides	≤11	280 (2.2)	0.10^a^	≤11	≤11	0.00
Amylin	≤11	≤11	−0.07	≤11	≤11	0.00
Metformin	193 (18.7)	3574 (27.6)	0.21^a^	189 (18.7)	182 (18.0)	−0.02
Aldosterone receptor antagonists	67 (6.5)	877 (6.8)	−0.01	65 (6.4)	64 (6.3)	0.00
α-Glucosidase inhibitor	≤11	66	0.02	≤11	≤11	0.01
Insulin pump	47 (4.6)	24 (0.2)	−0.29^a^	29 (2.9)	22 (2.2)	−0.04
Diabetes and ADRD events						
Hypoglycemia	264 (25.5)	1383 (10.7)	−0.39^a^	252 (24.9)	236 (23.3)	−0.04
Hypoglycemia hospitalizations	29 (2.8)	213 (1.6)	−0.08	29 (2.9)	27 (2.7)	−0.01
Hyperglycemia crisis	56 (5.4)	369 (2.8)	−0.13^a^	54 (5.3)	54 (5.3)	0.00
Diabetic neuropathy	329 (31.8)	3291 (25.4)	−0.14^a^	321 (31.8)	315 (31.2)	−0.01
Diabetic retinopathy	226 (20.7)	1341 (10.3)	−0.28^a^	212 (19.9)	358 (17.9)	−0.02
Diabetic foot ulcer	78 (7.5)	765 (5.9)	−0.07	78 (7.7)	61 (6.0)	−0.07
Diabetic cataract	≤11	125 (0.9)	0.00	≤11	≤11	0.00
Falls	106 (10.2)	1278 (9.9)	−0.01	103 (10.2)	117 (11.6)	0.04
Fracture	19 (1.8)	393 (3.0)	0.08	19 (1.9)	19 (1.9)	0.00
Syncope	132 (12.8)	1614 (12.5)	−0.01	132 (13.1)	134 (13.2)	0.01
Delirium	20 (1.9)	292 (2.2)	0.02	20 (1.9)	23 (2.3)	0.02
Healthcare utilization						
No. of primary care physician visits, mean (SD)	1.2 (3.3)	1.3 (3.7)	−0.03	1.2 (3.2)	1.2 (2.7)	0
No. of endocrinologist visits, mean (SD)	0.2 (0.9)	0.04 (0.4)	0.27^a^	0.2 (0.9)	0.2 (0.9)	0.01
No. of neurologist visits, mean (SD)	0.2 (0.9)	0.1 (0.5)	0.12^a^	0.2 (0.9)	0.1 (1.1)	0.02
Home health agency	380 (36.8)	6512 (50.3)	0.27^a^	374 (36.7)	364 (36.0)	−0.02
Skilled nursing facility	155 (14.9)	2761 (21.3)	0.16^a^	153 (15.1)	143 (14.1)	−0.03
No. of inpatient visits						
0-2	319 (30.8)	4118 (31.8)	0.02	310 (30.7)	310 (30.7)	0
3-4	257 (24.6)	3086 (23.8)	−0.02	247 (24.4)	258 (25.5)	−0.03
≥5	458 (44.3)	5750 (44.4)	0.00	454 (44.9)	443 (43.8)	−0.02
No. of outpatient visits						
0-10	236 (22.8)	4029 (31.1)	0.19^a^	233 (23.1)	250 (24.7)	0.04
11-20	224 (21.7)	2667 (20.6)	−0.03	222 (21.9)	221 (21.9)	0.00
≥21	574 (55.5)	6258 (48.3)	−0.14^a^	556 (55.0)	540 (53.4)	−0.03
No. of emergency department visits						
0	353 (34.1)	4751 (36.7)	0.05	344 (34.0)	345 (34.1)	0
1-2	263 (25.4)	2819 (21.8)	−0.09	258 (25.5)	258 (25.5)	0
≥3	418 (40.4)	5384 (41.6)	0.02	409 (40.5)	408 (40.4)	0
Marker for healthy behavior, frailty score & Charlson Comorbidity Index						
Vaccinations	594 (57.5)	5368 (41.4)	−0.32^a^	575 (56.9)	557 (55.1)	−0.03
Frailty score, mean (SD)	0.3 (0.1)	0.3 (0.1)	−0.08	0.3 (0.1)	0.3 (0.1)	0
Charlson Comorbidity Index, mean (SD)	5.8 (2.6)	5.6 (2.6)	0.05	5.8 (2.6)	5.8 (2.6)	−0.02

Abbreviations: ADRD, Alzheimer disease and related dementias; SMD, standardized mean difference.

^a^ Indicates imbalance (ie, SMD > 0.1).

^b^ Other indicates any race or ethnicity not otherwise specified.

^c^ Cross-suppressed to prevent recalculation of suppressed counts.

**Figure 2.**
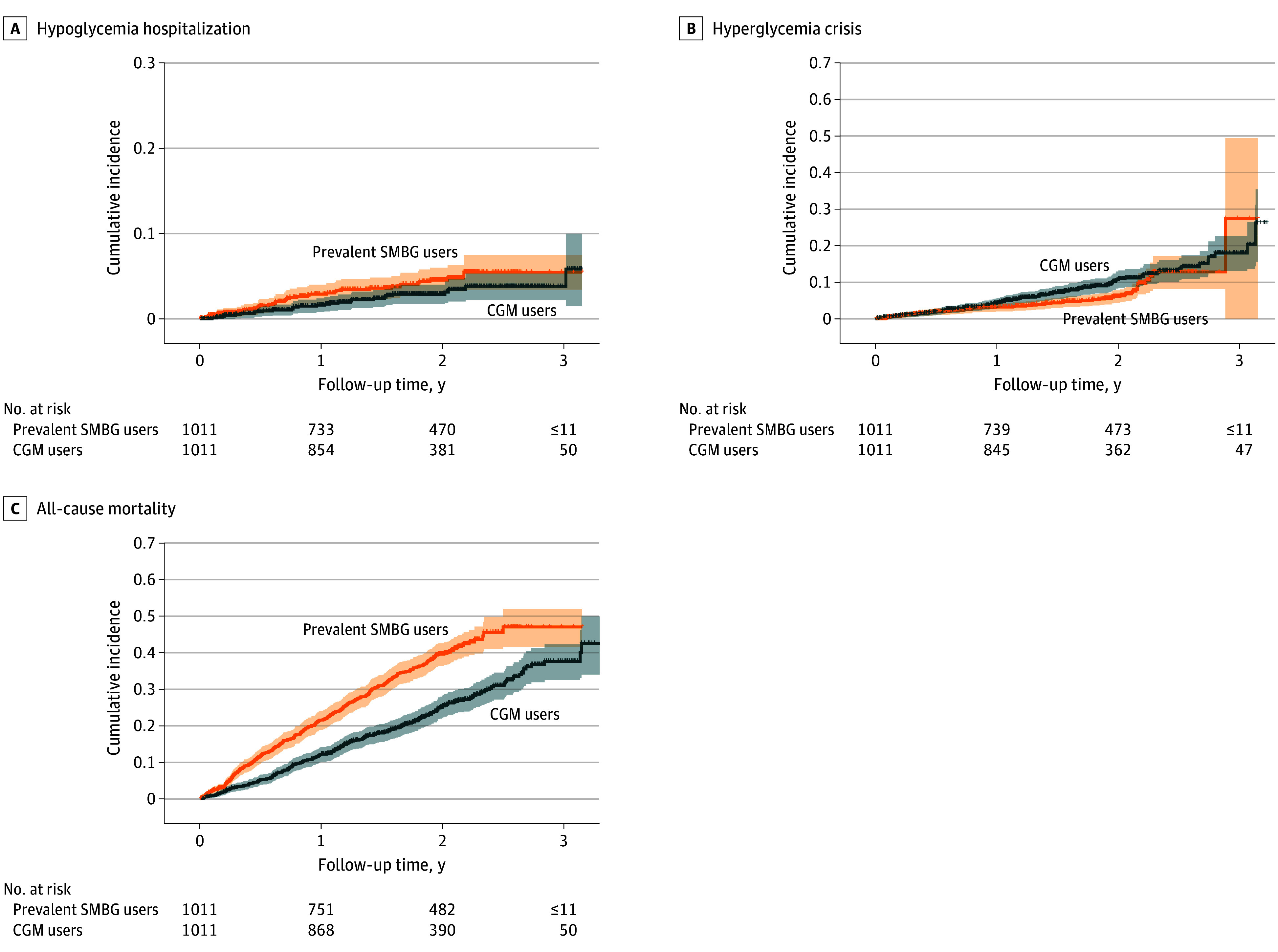
Cumulative Incidence of Primary Outcomes in a 1:1 Matched Cohort of Continuous Glucose Monitoring (CGM) Users vs Prevalent Self-Monitoring of Blood Glucose (SMBG) Users

**Figure 3.**
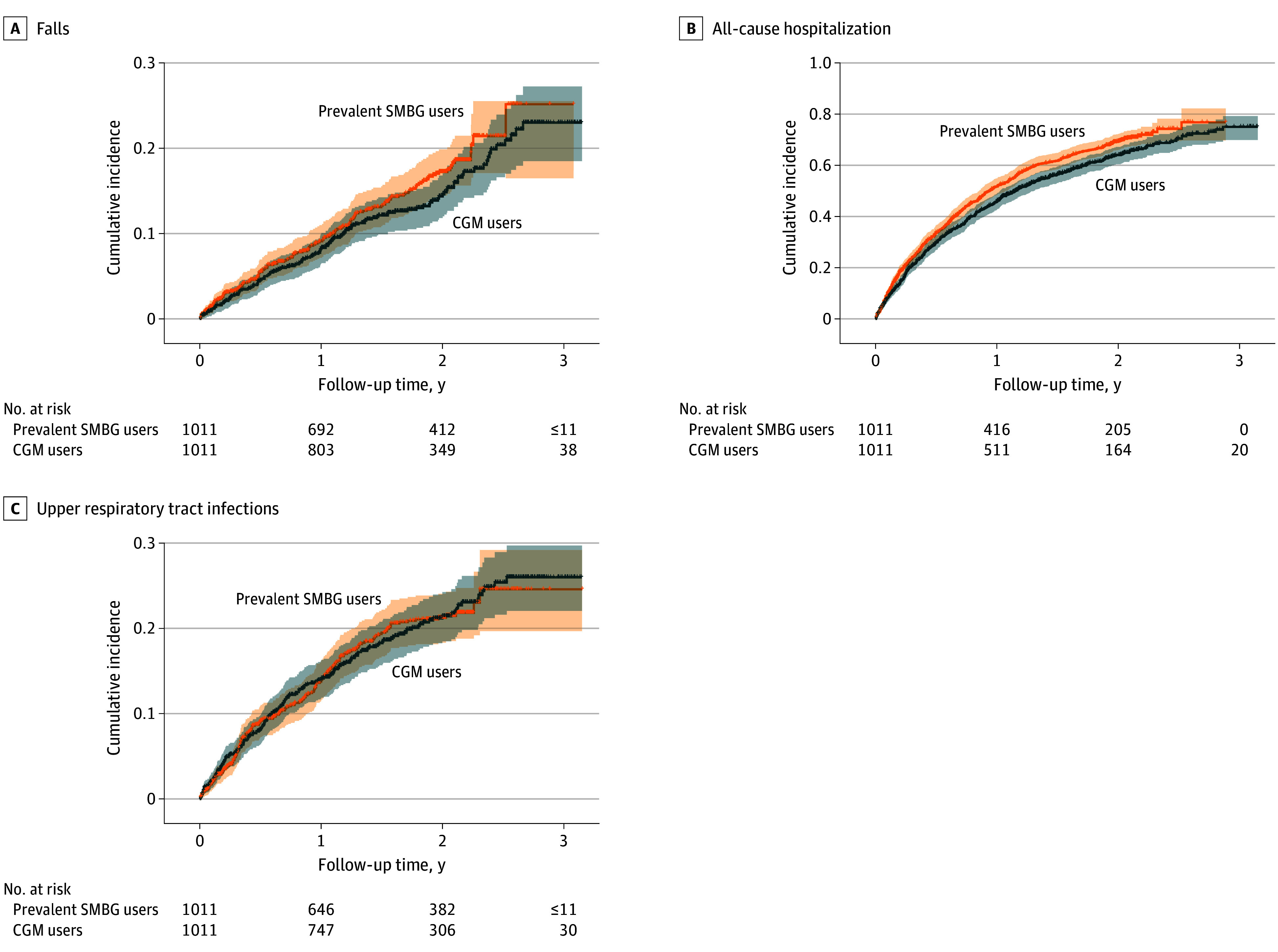
Cumulative Incidence of Secondary and Negative Control Outcomes in a 1:1 Matched Cohort of Continuous Glucose Monitoring (CGM) Users vs Prevalent Self-Monitoring of Blood Glucose (SMBG) Users

In the matched cohort, CGM use was associated with a significantly lower risk of all-cause hospitalizations (HR, 0.86; 95% CI, 0.76-0.96) and all-cause mortality (HR, 0.57; 95% CI, 0.48-0.67) compared with SMBG use. There were no significant differences in the risk of hypoglycemia hospitalizations (HR, 0.66; 95% CI, 0.40-1.08), hyperglycemia crisis (HR, 1.38; 95% CI, 0.99-1.94), or falls (HR, 0.86; 95% CI, 0.68-1.08) between CGM users and SMBG users (Figure 2 and Figure 3). The negative control outcome of upper respiratory tract infections showed no significant differences between the 2 groups (Figure 3). The number of events for outcomes and the incidence rate per 1000 person-years from the main analysis can be found in Table 2.

**Table 2.** Event Counts and Incidence Rates in CGM Users and SMBG Users for Main Analysis

Outcome	Event Counts, No. (%) (N = 2022)		Incidence rate per 1000 person-years	
	CGM users (n = 1011)	SMBG users (n = 1011)	CGM users	SMBG users
Hypoglycemia hospitalizations	29 (2.9)	38 (3.8)	16.77	24.24
Hyperglycemia crises	97 (9.6)	60 (5.9)	57.02	37.9
All-cause mortality	254 (25.1)	399 (39.5)	144.70	249.18
Falls	140 (13.8)	144 (14.2)	85.89	97.93
All-cause hospitalization	612 (60.5)	638 (63.1)	557.89	653.30
Upper respiratory tract infections	193 (19.1)	176 (17.4)	127.10	126.32

Abbreviations: CGM, continuous glucose monitoring; SMBG, self-monitoring of blood glucose.

Interactions with CGM use by sex, race, T1D, CCI, and frailty score were tested across outcomes, with significant interactions observed for T1D (*P *for interaction = .03) and frailty score (*P *for interaction = .04) for the hypoglycemia hospitalization outcome, T1D (*P* for interaction = .002) for the hyperglycemic crises outcome, frailty score (*P *for interaction = .02) for the falls outcome, CCI (P for interaction < .001) and frailty score (*P *for interaction < .001) for the all-cause hospitalizations outcome, and non-Hispanic White race (*P *for interaction = .02), CCI (*P *for interaction = .01), and frailty score (*P *for interaction < 0.001) for the mortality outcome.

In subgroup analysis, CGM use was associated with a significantly lower risk of hypoglycemia hospitalization in male CGM users compared with male SMBG users (HR, 0.35; 95% CI, 0.15-0.87), but not in female users (HR, 0.92, 95% CI, 0.50-1.70) or other subgroups. Conversely, CGM use was associated with a higher risk of hyperglycemia in T1D users (HR, 1.78; 95% CI, 1.15-2.75) and those with severe CCI scores (HR, 1.53; 95% CI, 1.02-2.29), with no significant differences in other subgroups. CGM use was also associated with lower all-cause mortality across most subgroups, except non-Hispanic Black and Asian and Pacific Islander individuals. Additionally, CGM use was associated with a lower risk of falls in patients with T1D (HR, 0.61; 95% CI, 0.42-0.91) and reduced all-cause hospitalizations in patients T1D (HR, 0.83; 95% CI; 0.69-0.99) and non-Hispanic White patients (HR, 0.86; 95% CI, 0.75-0.97). Sensitivity analysis for competing risks showed that CGM use was associated with a higher hyperglycemia risk (HR, 1.62; 95% CI, 1.17-2.24); there were no significant difference in risks of of hypoglycemia hospitalizations, falls, and hospitalizations, and 90-day hypoglycemia hospitalizations. Higher rates of 90-day hyperglycemic crises were observed in CGM users compared with SMBG users. (eFigure 4 in Supplement 1) The negative control outcome of upper respiratory tract infections showed no differences across all analyses, supporting current findings. (Figure 2 and Figure 3).

## Discussion

In this nationally representative cohort study using Medicare 2016 to 2020 data, CGM use was associated with a lower risk of all-cause hospitalization and mortality compared with prevalent SMBG users in insulin-treated older adults with diabetes and ADRD. Exploratory subgroup analysis revealed that CGM use was associated with a reduced risk of hypoglycemia hospitalization in male users, a lower risk of falls in patients with T1D, and a decreased risk of all-cause hospitalization in both T1D and non-Hispanic White subgroups, compared with prevalent SMBG users. Our study provides evidence supporting the clinical benefits of CGM use in potentially improving health outcomes for this vulnerable population.^8,9^

CGM use was associated with lower all-cause mortality compared with SMBG in the main, subgroup, and sensitivity analyses. These findings align with our pilot study^26^ in insulin-treated patients with diabetes and cognitive impairment, which showed significantly lower mortality in CGM users (adjusted odds ratio, 0.20; 95% CI, 0.10-0.60) and with the study by Reaven et al,^49^ reporting 14% (T2D) and 57% (T1D) lower mortality in CGM users. The Action to Control Cardiovascular Risk in Diabetes trial found that undetected hypoglycemia increased mortality risk.^50^ While we were unable to assess how many hypoglycemia episodes were undetected through retrospective data, we hypothesize that CGM alerts the patient or caregiver about the real-time glucose levels, promoting frequent reporting of hypoglycemia, leading to timely treatment and adjustments in dosage regimens compared with SMBG users, who may be unaware of their glycemic status, leading to a fatal outcome.

CGM use was associated with a lower risk of all-cause hospitalization compared with SMBG in the main analysis, consistent across most subgroups. This finding was aligned with findings from Reaven et al,^51^ in which CGM use was associated with lower hospitalization risk in patients with T1D (HR, 0.75; 95% CI, 0.63-0.90) and T2D (HR, 0.89; 95% CI, 0.82-0.97) compared with SMBG users.

CGM use was not associated with reduced risk of hypoglycemia hospitalizations overall, although the point estimates indicated a decreased rate; however, in subgroup analysis, males had a significantly lower risk of hypoglycemia hospitalizations. These differences by sex could be explained by prior research, which suggests women with diabetes are more prone to severe and nocturnal hypoglycemia, as well as heightened anxiety or fear of hypoglycemia, possibly leading to healthy-user behaviors and influencing CGM use and outcomes.^52,53^ Our findings were consistent with a previous study, which demonstrated there was no significant difference in hyperglycemia risk between CGM users and SMBG users, and similarly, they found that CGM use was associated with an increased risk of hyperglycemia in patients with T1D compared with SMBG users.^51^

To our knowledge, this is the first nationally representative study to assess the use of CGM in insulin-treated older adults with ADRD and diabetes. Our study bridged 4 subsections of diabetes care guidelines (Sections 13.5, 13.6, 13.8b, and 13.8c),^8^ emphasizing the need to connect these subsections for the diabetes treatment for patients with cognitive decline. We also integrated multiple recommendations from various professional societies into one comprehensive analysis, and the findings from this study can guide clinicians in treating this vulnerable population and inform treatment decisions. Our study also addresses the need suggested by several studies for further research to understand the long-term benefits of CGM use in patients with diabetes and cognitive impairment.^54,55,56^

### Limitations

However, our study has limitations. First, analyses were limited to Medicare fee-for-service beneficiaries, so findings may not generalize to managed care or privately insured populations. Second, exposure was determined from claims data; actual CGM or SMBG use was unknown, and some patients may have used CGM out of pocket or treated hypoglycemia at home with glucagon. Third, subgroup analyses were exploratory for hypothesis generation; results require cautious interpretation, and validation via future studies with larger sample sizes is needed. Fourth, claims data lacked key measures such as cognitive scores, hemoglobin A_1C_, and the extent of social support; these factors play an important role in patients’ outcomes and physicians’ choice to prescribe CGM. Although we addressed the lack of these direct measurements in several ways: baseline glycemic episodes, physician visits, support in the form of skilled nursing facility stays, and ADRD duration before CGM initiation, and SMBG dates to account for ADA guidelines regarding hypoglycemia management by cognitive function. The absence of direct measurement of these factors in the claims data should be accounted for while interpreting our findings. *ICD-10* codes for cognitive impairment severity (mild, moderate, or severe) were introduced in 2023, but were unavailable during 2016 to 2020, limiting severity assessment. Wider adoption of new *ICD-10* codes could improve specificity in future research.

## Conclusions

In conclusion, our findings suggest that CGM use was associated with better long-term outcomes in older adults with diabetes and ADRD compared with SMBG use. Due to the inherent limitations of claims-based observational studies, especially the lack of glycemic data and the level of social support, pragmatic (ie, evaluating the effectiveness of healthcare interventions in everyday settings) clinical trials are needed in the future to confirm the efficacy and feasibility of implementing CGM in patients with diabetes and ADRD. This study highlights the need for improved diabetes management for this particularly vulnerable patient population.

## Notes

Supplement 1. eFigure 1. Sample Illustration of Logic for Assigning Index Date to Prevalent SMBG Users eFigure 2. Distribution of Index Dates Since First ADRD Diagnosis in Prevalent SMBG Users and CGM Users eFigure 3. Propensity Score Distribution Before and After Matching eFigure 4. Subgroup and Sensitivity Analysis by Primary, Secondary, and Negative Control Outcomes

Supplement 2. Data Sharing Statement

## References

[1] U.S. Census Bureau. 2023 national population projections tables: main series. Updated February 12, 2025. Accessed October 3, 2025. https://www.census.gov/data/tables/2023/demo/popproj/2023-summary-tables.html

[2] Centers for Disease Control and Prevention. Chronic disease indicators: older adults. Updated June 3, 2024. Accessed October 3, 2025. https://www.cdc.gov/cdi/indicator-definitions/older-adults.html

[3] Zamrini E, Parrish JA, Parsons D, Harrell LE. Medical comorbidity in Black and White patients with Alzheimer’s disease. South Med J. 2004;97(1):2-6. doi:10.1097/01.SMJ.0000077061.01235.42 14746413

[4] Schubert CC, Boustani M, Callahan CM, . Comorbidity profile of dementia patients in primary care: are they sicker? J Am Geriatr Soc. 2006;54(1):104-109. doi:10.1111/j.1532-5415.2005.00543.x 16420205

[5] Yaffe K, Falvey CM, Hamilton N, ; Health ABC Study. Association between hypoglycemia and dementia in a biracial cohort of older adults with diabetes mellitus. JAMA Intern Med. 2013;173(14):1300-1306. doi:10.1001/jamainternmed.2013.6176 23753199 PMC4041621

[6] Punthakee Z, Miller ME, Launer LJ, ; ACCORD Group of Investigators; ACCORD-MIND Investigators. Poor cognitive function and risk of severe hypoglycemia in type 2 diabetes: post hoc epidemiologic analysis of the ACCORD trial. Diabetes Care. 2012;35(4):787-793. doi:10.2337/dc11-1855 22374637 PMC3308284

[7] Bruce DG, Davis WA, Casey GP, . Severe hypoglycaemia and cognitive impairment in older patients with diabetes: the Fremantle diabetes study. Diabetologia. 2009;52(9):1808-1815. doi:10.1007/s00125-009-1437-1 19575177

[8] American Diabetes Association Professional Practice Committee. 13. Older adults: standards of care in diabetes—2025. Diabetes Care. 2025;48(1)(suppl 1):S266-S282. 39651977 10.2337/dc25-S013 PMC11635042

[9] American Diabetes Association Professional Practice Committee. 4. Comprehensive medical evaluation and assessment of comorbidities: standards of care in diabetes—2025. Diabetes Care. 2025;48(1)(suppl 1):S59-S85. 39651988 10.2337/dc25-S004 PMC11635044

[10] Handelsman Y, Bloomgarden ZT, Grunberger G, . American association of clinical endocrinologists and american college of endocrinology - clinical practice guidelines for developing a diabetes mellitus comprehensive care plan - 2015. Endocr Pract. 2015;21(Suppl 1)(suppl 1):1-87. doi:10.4158/EP15672.GLSUPPL 25869408 PMC4959114

[11] Moreno G, Mangione CM, Kimbro L, Vaisberg E; American Geriatrics Society Expert Panel on Care of Older Adults with Diabetes Mellitus. Guidelines abstracted from the American Geriatrics Society Guidelines for improving the care of older adults with diabetes mellitus: 2013 update. J Am Geriatr Soc. 2013;61(11):2020-2026. doi:10.1111/jgs.12514 24219204 PMC4064258

[12] Lipska KJ, Ross JS, Miao Y, Shah ND, Lee SJ, Steinman MA. Potential overtreatment of diabetes mellitus in older adults with tight glycemic control. JAMA Intern Med. 2015;175(3):356-362. doi:10.1001/jamainternmed.2014.7345 25581565 PMC4426991

[13] Hopkins R, Shaver K, Weinstock RS. Management of adults with diabetes and cognitive problems. Diabetes Spectr. 2016;29(4):224-237. doi:10.2337/ds16-0035 27899874 PMC5111537

[14] Munshi MN, Segal AR, Suhl E, . Frequent hypoglycemia among elderly patients with poor glycemic control. Arch Intern Med. 2011;171(4):362-364. doi:10.1001/archinternmed.2010.539 21357814 PMC4123960

[15] Fløde M, Hermann M, Haugstvedt A, . High number of hypoglycaemic episodes identified by CGM among home-dwelling older people with diabetes: an observational study in Norway. BMC Endocr Disord. 2023;23(1):218. doi:10.1186/s12902-023-01472-6 37817166 PMC10566065

[16] Johansson M, Rogmark C, Sutton R, Fedorowski A, Hamrefors V. Risk of incident fractures in individuals hospitalised due to unexplained syncope and orthostatic hypotension. BMC Med. 2021;19(1):188. doi:10.1186/s12916-021-02065-7 34446019 PMC8394111

[17] Holden TR, Shah MN, Gibson TA, . Outcomes of patients with syncope and suspected dementia. Acad Emerg Med. 2018;25(8):880-890. doi:10.1111/acem.13414 29575587 PMC6156993

[18] Zhao Y, Kachroo S, Kawabata H, . Association between hypoglycemia and fall-related fractures and health care utilization in older veterans with type 2 diabetes. Endocr Pract. 2016;22(2):196-204. doi:10.4158/EP15640.OR 26492544

[19] Lee AK, Juraschek SP, Windham BG, . Severe hypoglycemia and risk of falls in type 2 diabetes: the atherosclerosis risk in communities (ARIC) study. Diabetes Care. 2020;43(9):2060-2065. doi:10.2337/dc20-0316 32611607 PMC7440903

[20] U.S. Centers for Disease Control and Prevention. Older Adult Falls Data. Updated October 28, 2024. Accessed October 3, 2025. https://www.cdc.gov/falls/data-research/index.html

[21] Lee AK, Warren B, Lee CJ, . The association of severe hypoglycemia with incident cardiovascular events and mortality in adults with type 2 diabetes. Diabetes Care. 2018;41(1):104-111. doi:10.2337/dc17-1669 29127240 PMC5741158

[22] Abdelhafiz AH, Rodríguez-Mañas L, Morley JE, Sinclair AJ. Hypoglycemia in older people—a less well recognized risk factor for frailty. Aging Dis. 2015;6(2):156-167. doi:10.14336/AD.2014.0330 25821643 PMC4365959

[23] Huang ES, Laiteerapong N, Liu JY, John PM, Moffet HH, Karter AJ. Rates of complications and mortality in older patients with diabetes mellitus: the diabetes and aging study. JAMA Intern Med. 2014;174(2):251-258. doi:10.1001/jamainternmed.2013.12956 24322595 PMC3950338

[24] Kagansky N, Levy S, Rimon E, . Hypoglycemia as a predictor of mortality in hospitalized elderly patients. Arch Intern Med. 2003;163(15):1825-1829. doi:10.1001/archinte.163.15.1825 12912719

[25] Li G, Zhong S, Wang X, Zhuge F. Association of hypoglycaemia with the risks of arrhythmia and mortality in individuals with diabetes—a systematic review and meta-analysis. Front Endocrinol (Lausanne). 2023;14:1222409. doi:10.3389/fendo.2023.1222409 37645418 PMC10461564

[26] Kotecha P, Chen W, Donahoo WT, Jaffee M, Bian J, Guo J. Continuous glucose monitoring and all-cause mortality in insulin-using population with diabetes and cognitive impairment. Diabetes Obes Metab. 2024;26(10):4795-4798. doi:10.1111/dom.15842 39134460

[27] Voss EA, Boyce RD, Ryan PB, van der Lei J, Rijnbeek PR, Schuemie MJ. Accuracy of an automated knowledge base for identifying drug adverse reactions. J Biomed Inform. 2017;66:72-81. doi:10.1016/j.jbi.2016.12.005 27993747 PMC5316295

[28] Zafari Z, Park JE, Shah CH, . The state of use and utility of negative controls in pharmacoepidemiologic studies. Am J Epidemiol. 2024;193(3):426-453. doi:10.1093/aje/kwad201 37851862 PMC11484649

[29] Lipsitch M, Tchetgen Tchetgen E, Cohen T. Negative controls: a tool for detecting confounding and bias in observational studies. Epidemiology. 2010;21(3):383-388. doi:10.1097/EDE.0b013e3181d61eeb 20335814 PMC3053408

[30] Ganz DA, Esserman D, Latham NK, . Validation of a rule-based *ICD-10-CM* algorithm to detect fall injuries in medicare data. J Gerontol A Biol Sci Med Sci. 2024;79(7):glae096. doi:10.1093/gerona/glae096 38566617 PMC11167485

[31] Colacci M, Fralick J, Odutayo A, Fralick M. Sodium-glucose cotransporter-2 inhibitors and risk of diabetic ketoacidosis among adults with type 2 diabetes: a systematic review and meta-analysis. Can J Diabetes. 2022;46(1):10-15.e2. doi:10.1016/j.jcjd.2021.04.006 34116926

[32] Min L, Tinetti M, Langa KM, Ha J, Alexander N, Hoffman GJ. Measurement of fall injury with health care system data and assessment of inclusiveness and validity of measurement models. JAMA Netw Open. 2019;2(8):e199679. doi:10.1001/jamanetworkopen.2019.9679 31433480 PMC6707014

[33] Ginde AA, Blanc PG, Lieberman RM, Camargo CA Jr. Validation of *ICD-9-CM* coding algorithm for improved identification of hypoglycemia visits. BMC Endocr Disord. 2008;8:4. doi:10.1186/1472-6823-8-4 18380903 PMC2323001

[34] Jarosek S. Death information in the research identifiable Medicare data. Published November 9, 2022. Accessed October 3, 2023. https://resdac.org/articles/death-information-research-identifiable-medicare-data

[35] Centers for Medicare & Medicaid Services. Glucose Monitors L33822. Updated October 9, 2024. Accessed October 3, 2025. https://www.cms.gov/medicare-coverage-database/view/lcd.aspx?lcdid=33822

[36] Puckrein GA, Hirsch IB, Parkin CG, . Assessment of glucose monitoring adherence in Medicare beneficiaries with insulin-treated diabetes. Diabetes Technol Ther. 2023;25(1):31-38. doi:10.1089/dia.2022.0377 36409474

[37] Suissa S, Dell’Aniello S. Time-related biases in pharmacoepidemiology. Pharmacoepidemiol Drug Saf. 2020;29(9):1101-1110. doi:10.1002/pds.5083 32783283

[38] Zhou Z, Rahme E, Abrahamowicz M, Pilote L. Survival bias associated with time-to-treatment initiation in drug effectiveness evaluation: a comparison of methods. Am J Epidemiol. 2005;162(10):1016-1023. doi:10.1093/aje/kwi307 16192344

[39] Kumar A, Sidhu J, Lui F, Tsao JW. Alzheimer Disease. StatPearls Publishing; 2025. 29763097

[40] Shrank WH, Patrick AR, Brookhart MA. Healthy user and related biases in observational studies of preventive interventions: a primer for physicians. J Gen Intern Med. 2011;26(5):546-550. doi:10.1007/s11606-010-1609-1 21203857 PMC3077477

[41] Kim DH, Schneeweiss S, Glynn RJ, Lipsitz LA, Rockwood K, Avorn J. Measuring frailty in Medicare data: development and validation of a claims-based frailty index. J Gerontol A Biol Sci Med Sci. 2018;73(7):980-987. doi:10.1093/gerona/glx229 29244057 PMC6001883

[42] Kim DH, Gautam N. SAS programs—claims-based frailty index version 15. Harvard Dataverse. Updated May 22, 2021. Accessed October 3, 2025. https://dataverse.harvard.edu/dataset.xhtml?persistentId=doi:10.7910/DVN/HM8DOI

[43] Charlson ME, Pompei P, Ales KL, MacKenzie CR. A new method of classifying prognostic comorbidity in longitudinal studies: development and validation. J Chronic Dis. 1987;40(5):373-383. doi:10.1016/0021-9681(87)90171-8 3558716

[44] Austin PC. An introduction to propensity score methods for reducing the effects of confounding in observational studies. Multivariate Behav Res. 2011;46(3):399-424. doi:10.1080/00273171.2011.568786 21818162 PMC3144483

[45] D’Agostino RB Jr. Propensity score methods for bias reduction in the comparison of a treatment to a non-randomized control group. Stat Med. 1998;17(19):2265-2281. doi:10.1002/(SICI)1097-0258(19981015)17:19<2265::AID-SIM918>3.0.CO;2-B 9802183

[46] Nolan EK, Chen HY. A comparison of the Cox model to the Fine-Gray model for survival analyses of re-fracture rates. Arch Osteoporos. 2020;15(1):86. doi:10.1007/s11657-020-00748-x 32519193

[47] Kim DH, Glynn RJ, Avorn J, . Validation of a claims-based frailty index against physical performance and adverse health outcomes in the health and retirement study. J Gerontol A Biol Sci Med Sci. 2019;74(8):1271-1276. doi:10.1093/gerona/gly197 30165612 PMC6625579

[48] Huang YQ, Gou R, Diao YS, . Charlson comorbidity index helps predict the risk of mortality for patients with type 2 diabetic nephropathy. J Zhejiang Univ Sci B. 2014;15(1):58-66. doi:10.1631/jzus.B1300109 24390745 PMC3891119

[49] Reaven PD, Macwan S, Newell M, . Initiation of continuous glucose monitoring and mortality in type 2 diabetes. Diabetes Technol Ther. 2025. doi:10.1089/dia.2025.0227 40432529 PMC13398955

[50] Seaquist ER, Miller ME, Bonds DE, ; ACCORD Investigators. The impact of frequent and unrecognized hypoglycemia on mortality in the ACCORD study. Diabetes Care. 2012;35(2):409-414. doi:10.2337/dc11-0996 22179956 PMC3263892

[51] Reaven PD, Newell M, Rivas S, Zhou X, Norman GJ, Zhou JJ. Initiation of continuous glucose monitoring is linked to improved glycemic control and fewer clinical events in type 1 and type 2 diabetes in the Veterans Health Administration. Diabetes Care. 2023;46(4):854-863. doi:10.2337/dc22-2189 36807492 PMC10260873

[52] Talbo MK, Lebbar M, Wu Z, . Gender differences in reported frequency and consequences of hypoglycemia among adults living with type 1 diabetes: results from the BETTER registry. Diabetes Res Clin Pract. 2023;202:110822. doi:10.1016/j.diabres.2023.110822 37423499

[53] Kautzky-Willer A, Kosi L, Lin J, Mihaljevic R. Gender-based differences in glycaemic control and hypoglycaemia prevalence in patients with type 2 diabetes: results from patient-level pooled data of six randomized controlled trials. Diabetes Obes Metab. 2015;17(6):533-540. doi:10.1111/dom.12449 25678212 PMC6680342

[54] Beck RW, Riddlesworth TD, Ruedy K, ; DIAMOND Study Group. Continuous glucose monitoring versus usual care in patients with type 2 diabetes receiving multiple daily insulin injections: a randomized trial. Ann Intern Med. 2017;167(6):365-374. doi:10.7326/M16-2855 28828487

[55] Beck RW, Riddlesworth T, Ruedy K, ; DIAMOND Study Group. Effect of continuous glucose monitoring on glycemic control in adults with type 1 diabetes using insulin injections: the DIAMOND randomized clinical trial. JAMA. 2017;317(4):371-378. doi:10.1001/jama.2016.19975 28118453

[56] Pratley RE, Kanapka LG, Rickels MR, ; Wireless Innovation for Seniors With Diabetes Mellitus (WISDM) Study Group. Effect of continuous glucose monitoring on hypoglycemia in older adults with type 1 diabetes: a randomized clinical trial. JAMA. 2020;323(23):2397-2406. doi:10.1001/jama.2020.6928 32543682 PMC7298607

