# Supplementary material for: Continuous Glucose Monitoring in Insulin-Treated Older Adults With Diabetes and Alzheimer Disease and Related Dementias
Source: JAMA Netw Open. 2025 Dec 2;8(12):e2541939. doi: 10.1001/jamanetworkopen.2025.41939 (PMC12673414; doi:10.1001/jamanetworkopen.2025.41939)
Supplement: Supplement 1. — eFigure 1. Sample Illustration of Logic for Assigning Index Date to Prevalent SMBG Users eFigure 2. Distribution of Index Dates Since First ADRD Diagnosis in Prevalent SMBG Users and CGM Users eFigure 3. Propensity Score Distribution Before and After Matching eFigure 4. Subgroup and Sensitivity Analysis by Primary, Secondary, and Negative Control Outcomes [file jamanetwopen-e2541939-s001.pdf]

Supplemental Online Content

Kotecha P, Smith SM, Donahoo WT, DeKosky ST, Bian J, Guo J. Continuous glucose monitoring outcomes in insulin-treated older adults with diabetes and alzheimer disease and related dementias. *JAMA Netw. Open*. 2025;8(11):e2541939. doi:10.1001/jamanetworkopen.2025.41939

eFigure 1. Sample Illustration of Logic for Assigning Index Date to Prevalent SMBG Users

eFigure 2. Distribution of Index Dates Since First ADRD Diagnosis in Prevalent SMBG Users and CGM Users

eFigure 3. Propensity Score Distribution Before and After Matching

eFigure 4. Subgroup and Sensitivity Analysis by Primary, Secondary, and Negative Control Outcomes

This supplemental material has been provided by the authors to give readers additional information about their work.

© 2025 Kotecha P et al. *JAMA Network Open*.

eFigure1 Sample illustration of logic for assigning index date to Prevalent SMBG Users

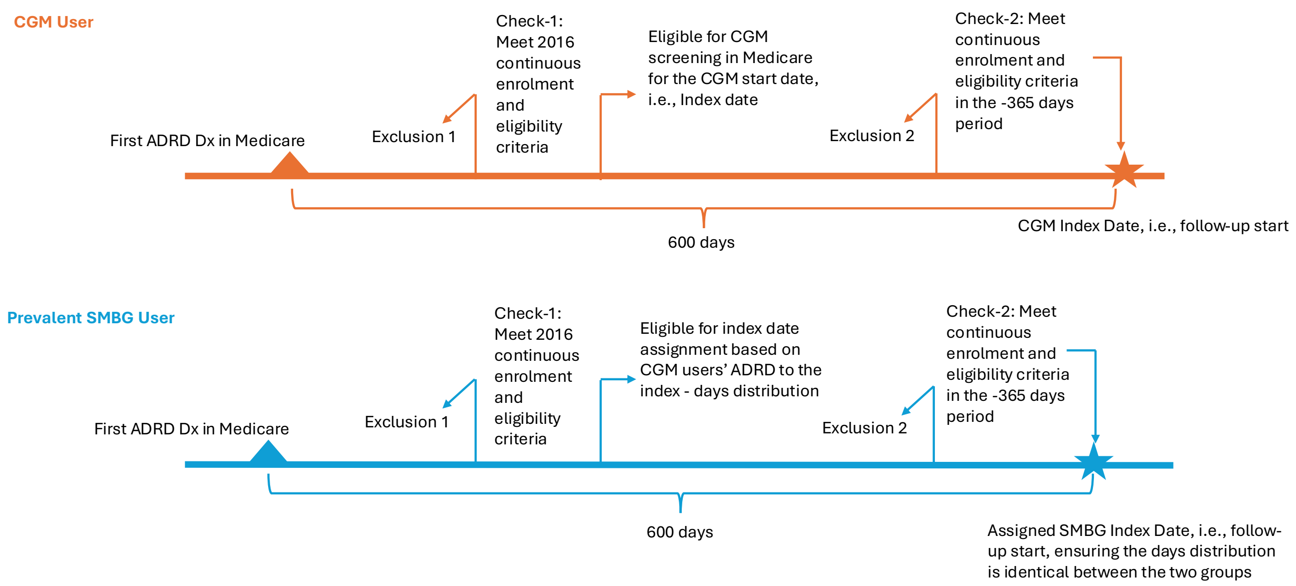

This illustration outlines how index dates were assigned for SMBG users. Initial eligibility was assessed in 2016, requiring a diabetes diagnosis, ADRD diagnosis, and an insulin claim, with exclusion of patients in hospice or without continuous enrollment. CGM use was then evaluated from 2017–2019; patients with a CGM claim were assigned the date of first CGM use as their index date. Patients without CGM during the study period but with SMBG use in 2016 were assigned a hypothetical index date, chosen to match the distribution of time from ADRD diagnosis to index date observed among CGM users. Eligibility was rechecked 365 days before the assigned/actual index date for both groups. Hypothetical index dates were on or after January 1, 2017, and before the end of continuous enrollment (continuous enrollment was assessed starting Jan 1, 2016).

© 2025 Kotecha P et al. *JAMA Network Open*.

eFigure2 Distribution of index dates since first ADRD diagnosis in Prevalent SMBG users and CGM users

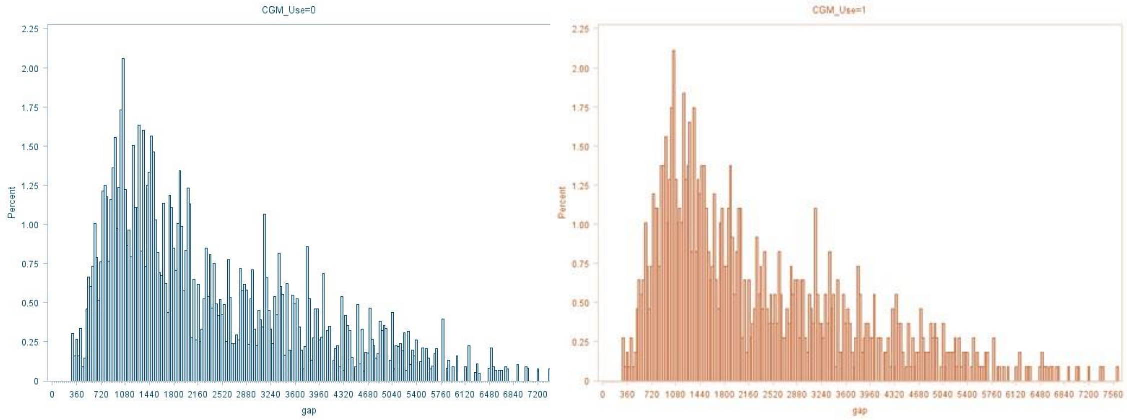

Wilcoxon two-sample test p=0.26.

© 2025 Kotecha P et al. *JAMA Network Open*.

eFigure3 Propensity Score Distribution Before and After Matching

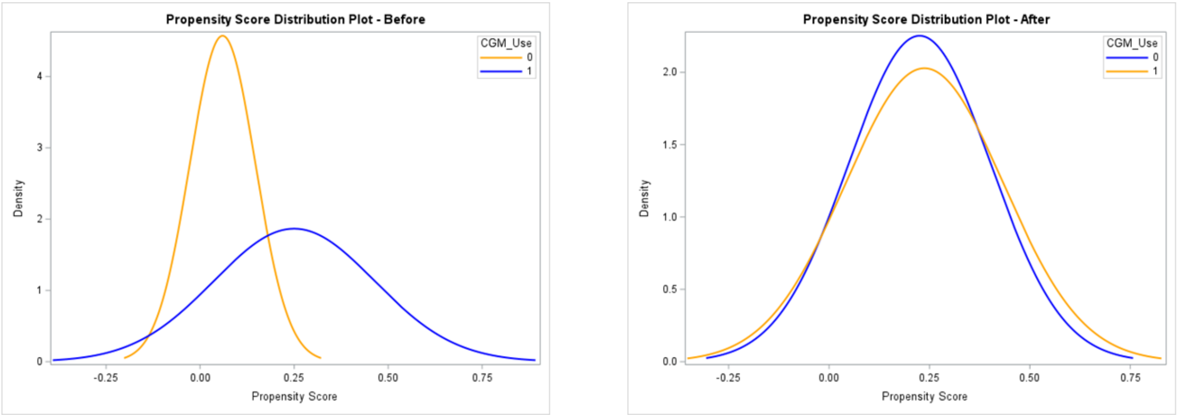

© 2025 Kotecha P et al. *JAMA Network Open*.

eFigure4 Subgroup and Sensitivity Analysis by Primary, Secondary and Negative Control Outcomes

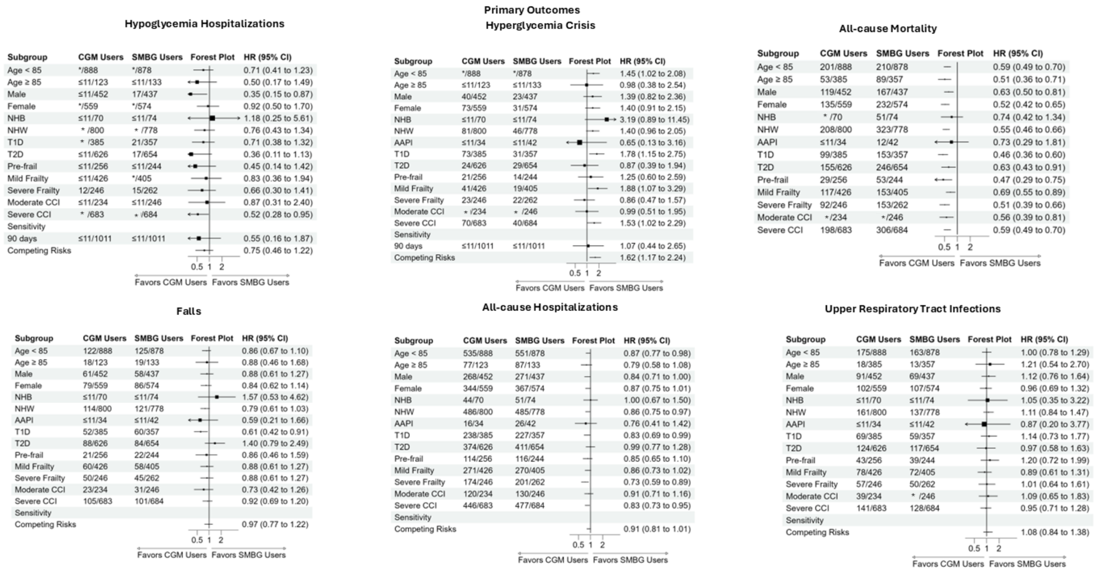

*not ≤11 but suppressed to mask other low counts

Footnote: *The AAPI group had few events, and HR (95% CI) could not be determined for hypoglycemia hospitalizations in this subgroup*.

Abbreviations: NHB: Non-Hispanic Black, NHW: Non-Hispanic White, AAPI: Asian American and Pacific Islander, T1D: Type 1 diabetes, T2D: Type 2 diabetes, CCI: Charlson Comorbidity Index

© 2025 Kotecha P et al. *JAMA Network Open*.
