# Supplementary material for: Continuous Glucose Monitoring in Insulin-Treated Older Adults With Diabetes and Alzheimer Disease and Related Dementias
Source: JAMA Netw Open. 2025 Dec 2;8(12):e2541939. doi: 10.1001/jamanetworkopen.2025.41939 (PMC12673414; doi:10.1001/jamanetworkopen.2025.41939)
Supplement: Supplement 2. — Data Sharing Statement [file jamanetwopen-e2541939-s002.pdf]

Data Sharing Statement

Kotecha. Continuous Glucose Monitoring in Insulin-Treated Older Adults With Diabetes and Alzheimer Disease and Related Dementias. *JAMA Netw Open*. Published November 05, 2025. doi:10.1001/jamanetworkopen.2025.41939

Data
Data available: No

Additional Information
Explanation for why data not available: The data analyzed in this study are subject to the following licenses/restrictions: The Medicare claims data contain patient-level health information and are considered identifiable files. Therefore, access to these data requires a data use agreement. Requests to access these datasets should be directed to resdac@umn.edu.
